# Bone regeneration using composite non-demineralized xenogenic dentin with beta-tricalcium phosphate in experimental alveolar cleft repair in a rabbit model

**DOI:** 10.1186/s12967-017-1369-3

**Published:** 2017-12-23

**Authors:** Mohammad Kamal, Lars Andersson, Rene Tolba, Adel Al-Asfour, Alexander K. Bartella, Felix Gremse, Stefanie Rosenhain, Frank Hölzle, Peter Kessler, Bernd Lethaus

**Affiliations:** 1 Department of Cranio-Maxillofacial Surgery and GROW School for Oncology and Developmental Biology, Maastricht University Medical Center, P. Debyelaan, Postbus 5800, 6202 AZ Maastricht, The Netherlands; 2 Department of Oral and Maxillofacial Surgery, RWTH Aachen University, Pauwelsstraße 30, 52074 Aachen, Germany; 3 Department of Surgical Sciences, Health Sciences Center, Kuwait University, 13110 Safat, Kuwait; 4 Institute for Laboratory Animal Science and Experimental Surgery, RWTH Aachen University, Pauwelsstraße 30, 52074 Aachen, Germany; 5 Department of Experimental Molecular Imaging, RWTH Aachen University, Pauwelsstraße 30, 52074 Aachen, Germany

**Keywords:** Animal testing, Cleft lip and palate, Alveolar cleft, Bone transplantation, Grafting, Tissue-engineering, Dentin, Rabbit

## Abstract

**Background:**

Alveolar cleft repair is performed via bone grafting procedure to restore the dental arch continuity. A suitable bone substitute materials should possess osteoinductive and osteoconductive properties, to promote new bone formation, along with a slowly resorbable scaffold that is subsequently replaced with functionally viable bone. Calcium phosphate biomaterials have long proved their efficacy as bone replacement materials. Dentin in several forms has also demonstrated its possibility to be used as bone graft replacement material in several studies. The purpose of this study was to evaluate bone regeneration pattern and quantify bone formation after grafting pre-established experimental alveolar clefts defects model in rabbits using composite xenogenic dentin and β-TCP in comparison to β-TCP alone.

**Methods:**

Unilateral alveolar cleft defects were created in 16 New Zealand rabbits according to previously described methodology. Alveolar clefts were allowed 8 weeks healing period. 8 defects were filled with β-TCP, whereas 8 defects filled with composite xenogenic dentin with β-TCP. Bone regeneration of the healed defects was compared at the 8 weeks after intervention. Quantification of bone formation was analyzed using micro-computed tomography (µCT) and histomorphometric analysis.

**Results:**

µCT and histomorphometric analysis revealed that defects filled with composite dentin/β-TCP showed statistically higher bone volume fraction, bone mineral density and percentage residual graft volume when compared to β-TCP alone. An improved surgical handling of the composite dentin/β-TCP graft was also noted.

**Conclusions:**

Composite xenogenic dentin/β-TCP putty expresses enhanced bone regeneration compared to β-TCP alone in the reconstruction of rabbit alveolar clefts defects.

## Background

Maxillary alveolar cleft is a congenital malformation occuring during fetal development leading to a compromised dental arch anatomy and disrupted sequence teeth eruption. Alveolar cleft repair is performed during childhood via bone grafting procedure with the goal to restore the bony continuity of the dental arch, seal the oronasal communication, and create a favorable anatomy for dental rehabilitation [1–5]. Alveolar bone cleft is routinely grafted with autogenous bone obtained from various anatomical donor sites; however, autogenous bone is associated with inherent technical limitations such as donor site supply limitation and surgical morbidity, increased operative time and costs, and several clinical reports on its increased resorption rate with unpredictable graft stability [5–12].

To overcome these shortcomings with autogenous bone, various regenerative bone substitute materials have been developed for skeletal grafting including processed xenogeneic bone [13], allogenic bone [14, 15], several biodegradable alloplastic materials such as polymer based polyether ketone and fiber-reinforced bioactive glass materials [16, 17], and various degradable bioactive ceramics like tricalcium phosphate and hydroxyapatite [6, 9, 11, 12, 15, 18, 19].

Biocompatible calcium phosphate ceramics have shown in several human and animal studies to express osteoconductive properties leading to enhanced bone formation and the ability to function as a bone grafting substitute materials [3, 14, 15, 17–20]. Several types of calcium phosphate currently exist for clinical applications, with biphasic beta tri-calcium phosphate (β-TCP) and calcium hydroxyapatite (HA) being the most common utilized. β-TCP and HA are mixed in a proportionate manner to combine the resiliency and improved mechanical properties offered by hydroxyapatite (HA), along with the reliable biodegradability offered by tri-calcium phosphate (β-TCP) to yield a suitable bone replacement materials with balanced clinical efficacy [18, 20].

Dentin, a natural hard tissue component of teeth and similar in its chemical composition to bone, has likewise been explored as a bone replacement material. Several human and in vivo studies have demonstrated the ability of dentin to promote bone formation due to its innate osteoconductive and osteoinductive properties [13, 21–28]. The osteoconductive potential of dentin was evaluated in its non-demineralized form in an animal model by implanting 2–3 mm dentin blocks into bone marrow of rabbits, and subsequently leading bone formation close to the native tibia bone wall indicating the ability of the dentin blocks to induce osteogenesis [21]. In addition to its known clinical effect in promoting bone formation and fusing with bone in a process of ankylosis as seen in several studies evaluating the replantation of avulsed teeth in human without the process of demineralization [23, 24, 29–31]. Several studies also demonstrated such osteoinductivity potential of dentin when used in demineralized form, however, higher degree of dentin resorption rate and thus lower graft volume maintenance rate was report in these studies [22, 27, 32, 33]. Dentin has also been studied in various allogenic and xenogenic forms to explore its efficacy as bone replacement material including mineralized dried dentin as particulate chips and blocks, demineralized dentin, freeze-dried allogenic dentin, and processed bovine dentin [13, 22, 25–28, 34].

Despite recent biotechnological developments of bone substitute materials, optimising the quality of the existing regenerative materials and looking for novel and more effective materials is essential in developing a clinically suitable material. The establishment of a proper in vivo biological model simulating alveolar clefts is essential in order to conduct experimental testing of bone grafting materials and evaluate the clinical effect with respect to osteogenesis and healing, and thus far several animal models have been developed in mice, rats, rabbits, cats, dogs, swines, goats, sheep and monkeys [2, 6, 35–49].

In a recent paper, an in vivo rabbit model with surgically created alveolar clefts for the clinical testing was presented [45]. The advantage with this model is that the cleft is kept open during the healing to avoid rapid bone generation and filling of the defect, enabling conditions similar to clefts seen in clinical practice [50].

Giving the previously reported healing capacity of non-demineralized dentin when used as bone replacement material, we wanted to investigate the effect of combining non-demineralized dentin with the well-established β-TCP, and report on any enhancement in bone volume fraction, which may have caused by the adding dentin to β-TCP. We propose that adding dentin would enhance the overall bone formation in the defect, including the fusion of the dentin particles to the healed graft though osseos integration with the newly formed bone, and undergoing a gradual and slow resorption process, thus maintaining a higher BV/TV ratio and improved graft volume maintenance rate. The aim of the current study was to evaluate bone regeneration potential and quantify the overall bone formation after grafting pre-established experimental alveolar clefts defects in a rabbit model using in situ formed composite xenogenic non-demineralized dentin and β-TCP in comparison to β-TCP alone.

## Methods

### Animals, anesthesia, and housing

**Fig. 1:**
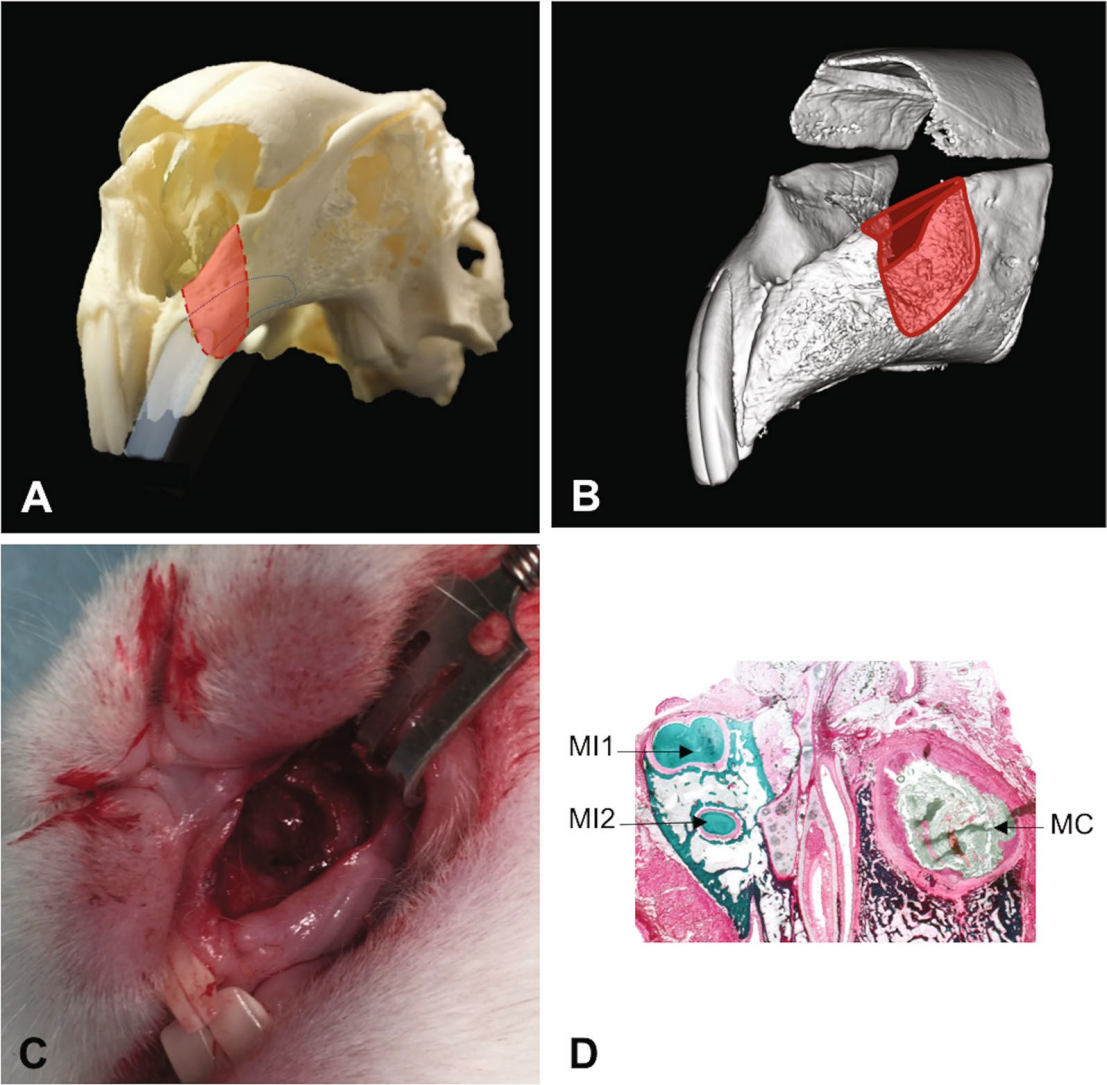
Alveolar cleft model. **A** Osteological representation of the created alveolar cleft extending to the nasal mucosa on a New Zealand White rabbit skull. **B** Micro-CT imaging of the created cleft 8 weeks after cleft creation surgery representing the three-dimensional morphology of the healed residual defect. **C** Surgical exposure of the healed alveolar cleft for insertion of the grafting intervention 8 weeks after cleft creation surgery. **D** Coronal histological representation of the healed alveolar cleft 8 weeks after cleft creation surgery with cleft area fully lined by mucosal tissue (MI1, first maxillary incisor; MI2, second maxillary incisor; MC, maxillary cleft)

A New Zealand White rabbit model for alveolar cleft testing was previously described by the authors in detail (Fig. 1) [50]. The experimental procedure was conducted at the Animal Research Centre, Health Sciences Centre, Kuwait University. The project was subjected to strict animal testing protocol and the approval by the animal ethical committee at Animal Research Centre, Health Sciences Centre, Kuwait University. Sixteen 8-week-old New Zealand White rabbits weighing 2.6–3.0 kg were operated in the same manner to prove the in vivo model. The rabbits were sedated with Xylazine HCl 5 mg/kg by intramuscular injection 30 min prior to operation and subsequently anesthetized with 35 mg/kg of ketamine HCl by intravenous injection. To ascertain the highest standard of animal care, a veterinarian was administering the sedation, anesthesia, and care-taking of the animals according to previously used methodology [21, 25, 50]. Throughout the duration of the study, rabbits were kept in separate cags and fed rabbit pellets and water mixture, and were cared of accordingly and observed by veterinarian until the completion of the study.

### Preparation of grafting material

**Fig. 2:**
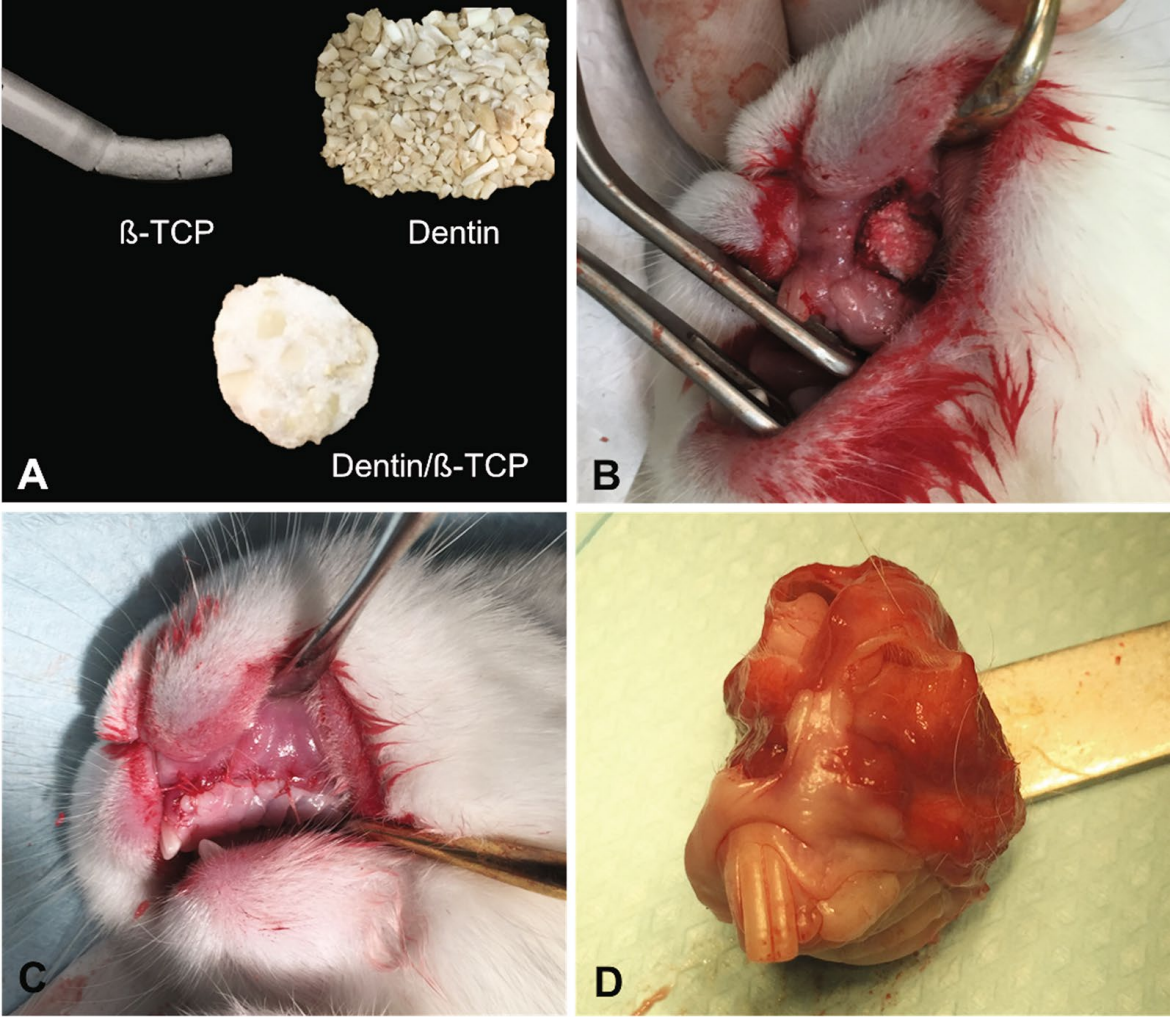
Graft preparation. **A** Injectable β-TCP/HA putty (Maxresorb inject, Botiss Biomaterials, Berlin, Germany), dentin blocks, and the in situ formed composite dentin/β-TCP mixture in preparation for grafting intervention. **B** Insertion of the grafting intervention in the alveolar cleft defect at 8 weeks after cleft creation surgery. **C** Tension-free closure of the oral mucosa over the grafted alveolar cleft defect. **D** Block sections of the maxilla specimens contained the repaired alveolar clefts 8 weeks after insertion of the grafting intervention

Human teeth, extracted for orthodontic reasons and then stored dry, were prepared by first removing the crowns then sectioning the roots vertically to expose the pulp and root canal. The periodontal ligament and pulp were removed mechanically, and dentin chips 2**–**3 mm in diameter were prepared by crushing the remaining dentin in a mortar and passing it through a 2-mm grid to maximize the size of the chips. The chips were cleaned from smaller dentin particles by soaking them in 1% chlorhexidine for 5 min and then stored dry. For the comparative grafting intervention, a commercially available hydroxyapatite/biphasic tricalcium phosphate injectable putty bone-substitute material made of 60% HA/40% β-TCP ratio (Maxresorb inject, Botiss Biomaterials, Berlin, Germany) was utilized and prepared per manufacturer’s protocol. Dentin particles and β-TCP/HA putty were subsequently mixed together intraoperatively by the same operator for all interventions to ensure consistency of preparation and volume proportion and to create a moldable grafting mixture (Fig. 2). The mixture was made using 0.5 cc of Maxresorb inject (60% hydroxyapatite and 40% β-tricalcium phosphate, Botiss Biomaterials, Berlin, Germany) with predetermined volume of dentin blocks obtained using the same dental scoop excavator.

### Preparation of surgical sites in maxillary alveolus

The surgery was conducted under sterile conditions with the rabbits in a supine position. A buccal gingival flap was raised subperiosteally to expose the periodontal attachment of the central incisor tooth and the inferior nasal aperture as previously described in our model [50]. Osteotomy was performed along the lateral curvature of the left central incisor using a rotary instrument to expose the root of the central incisor, then tooth was gently luxated laterally and extracted using extraction forceps. Further osteotomy was carried under protection of the nasal mucosa to remove the superior and inferior bony plates and create an oral-nasal defect with intact nasal mucosa. Bone wax (Ethicon Inc., Somerville, New Jersey, USA) then applied to the bony walls of the defect and the oral mucosa was approximated and sutured on the medial and lateral sides with 5 zeros resorbable Vicryl suture (Ethicon Inc., New Jersey, USA) leaving the central part of the wound open to create a pocket into the cleft defect, into which surgicel oxidised cellulose (Johnson & Johnson, New Brunswick, New Jersey, USA) was packed. The animals were allowed a period of 8 weeks for healing of the defect and the creation of maxillary alveolar defect [50].

### Exposure of the maxillary alveolus defects and insertion of the bone substitute materials

The same surgical preparation and the strict protocol was carried during the second surgery to expose the alveolar defect in preparation of bone grafting. An incision in the non-keratinized mucosa is made in the alveolar defect area to separate the oral from the nasal mucosa. The bony defect was exposed and bony walls were lightly freshened with a round diamond bur. In 8 rabbits the defects were filled with composite xenogenic dentin with beta-tricalcium phosphate (intervention), whereas in 8 rabbits the defects were filled with beta-tricalcium phosphate alone (control) as seen in Fig. 2. The grafting material was lightly placed into defect without compression. Primary wound closure was performed using 5 zeros resorbable Vicryl suture (Ethicon Inc., Somerville, New Jersey, USA). The rabbits were fed soft diet ad lib directly after the surgery. The rabbits were cared of accordingly per protocol and watched by veterinarian until the end of the study 3 months.

### Animal sacrifice and qualitative evaluation

Animals were sacrificed at 8 weeks postoperatively with an intravenous lethal dose of T61 Euthanasia Solution (Embutramide, Mebezonium iodide, Tetracaine hydrochloride) after sedation with an intramuscular injection of Xylazine HCl 5 mg/kg. Block sections of the maxilla specimens were obtained using oscillating saw maintaining the adjacent soft tissue and fixed in 10% neutral-buffered formalin (Fig. 2). The specimens were grossly inspected for inflammation. Specimen were subsequently scanned with µCT before histological processing. Healing of the biomaterial-filled defects was compared radiographically and by histomorphometry. Micro-computed tomography (µCT) was utilized to analyze the osteogenesis and healing patterns of the defects with different interventions. Quantitative analysis of bone mineral density (BMD) and bone volume fraction (%) of the new bone formation (BV = bone volume/TV = tissue volume) was evaluated in the defects filled with each group.

### Acquisition of micro-CT scans, segmentation, and statistical analysis

After animal sacrifice, the maxillas including the created alveolar defects with filling materials were harvested for ex vivo micro-CT imaging and histological analysis. To acquire the micro-CT images, maxillas were imaged ex vivo in μCT (Tomoscope 30 s Duo, CT-Imaging, Erlangen, Germany) according to a protocol previously described by authors [51]. After positioning in a multimodal holder, mandibles were scanned with the μCT. A dual energy scan (HQD-6565-360-29) which acquires 720 projections with 1032 × 1012 pixels with scanning time 90 s per subscan was used. To cover one maxilla, two subscans were acquired. Both tubes of the dual source µCT were operated with voltage of 65 kV and current of 1 mA. The μCT data was reconstructed at an isotropic voxel size of 35 μm using a Feldkamp type algorithm and a smooth kernel. For analysis, the μCT data was down-sampled using binning to a voxel size of 70 μm. To determine the bone mineral density score and the new formed bone volume the 70 µm µCT file was analyzed with the Imalytics Preclinical software [52]. To segment the alveolar cleft, scribbles were drawn slice-wise to delineate the boundaries of the fillings and newly formed bones. A threshold over 600 HU was applied to segment the bones inside the area. Relative bone volume and mean intensity (as bone density score) inside the segmented class were determined as volumetric ratio. As an added measure to reduce bias in the measurement readings of the defect size on µCT images, measurements of defect size determination were scored independently by two imaging specialists and confirmed by the operator. The statistical data was analyzed using GraphPad Prism (Version 6.0 for Windows, GraphPad Software, La Jolla, California, USA). To compare the measurements between TCP and dentin, unpaired t test was performed in combination with a Tukey post-test. A p value below 0.05 was considered to represent statistical significance.

### Histologic preparation histomorphometry, and statistical analysis

After µCT image acquisition, the specimens were dehydrated using ascending ethanol gradient (50–100%) prior to embedding in methylmethacrylate resin (Technovit 9100, Heraeus Kulzer GmbH, Frankfurt, Germany). Coronal sections of the embedded undecalcified specimens were obtained at a thickness of about 200 µm using the EXAKT cutting unit (EXAKT Technologies Inc., Oklahoma City, Oklahoma, USA), then thinned and polished manually to a final thickness of about 50–70 µm. Final specimens were stained with Masson’s trichrome (MTC) and toluidine blue (TB) according to protocol, and were analyzed using light microscopy. Three slides for each defect was obtained in the coronal section through the center grafted defect after visual inspection of the embedded specimens. The middle slice was selected for analysis in all the cases. Histomorphometric evaluation for performed with ImageJ software (ImageJ 1.51p, National Institute of Health, Bethesda, Maryland, USA) by manually measuring the compartments of new bone area, and remaining grafting materials. Mean percentage of bone formation per total defect volume (% = new bone area/total defect area) and standard deviation (SD) were used for each group. Statistical analysis conducted using GraphPad Prism (Version 6.0 for Windows, GraphPad Software, La Jolla, California, USA) using unpaired t test was performed in combination with a Tukey post-test. A p value below 0.05 was considered to represent statistical significance.

## Results

### Surgical procedure and experimental observations

All rabbits survived the surgical procedure. In a few cases an extra injection of Ketamine was required. Handling of the putty β-TCP/HA during surgery was very effective and facilitated the in situ mixing with dentin chips and insertion of the composite graft in the alveolar defect without the need of any additional handling, such as wetting or drying. The graft showed acceptable dimensional stability and retained in the defect adequately. Intraoperative and postoperative bleeding was minimal and did not result in any clinical issues. The animals were active and behaved adequately immediately after the surgery. They all started eating already during the first day after surgery. The animals were fed soft pellets ad libitum throughout the study and gained weight. All the rabbits survived during the 8 weeks postoperatively until day of the sacrifice.

### µCT imaging of the filled alveolar defects

**Fig. 3:**
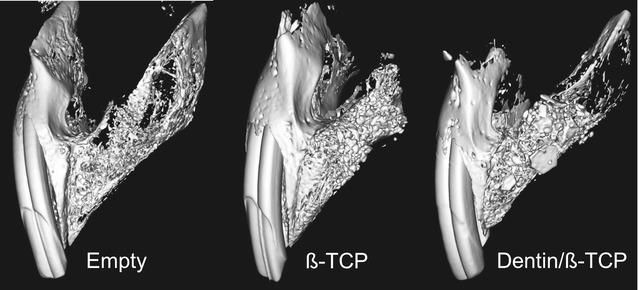
µCT imaging. µCT imaging reconstruction of the created alveolar clefts 8 weeks after cleft creation surgery, and 8 weeks after insertion of the grafting intervention with β-TCP and dentin/β-TCP composite

**Fig. 4:**
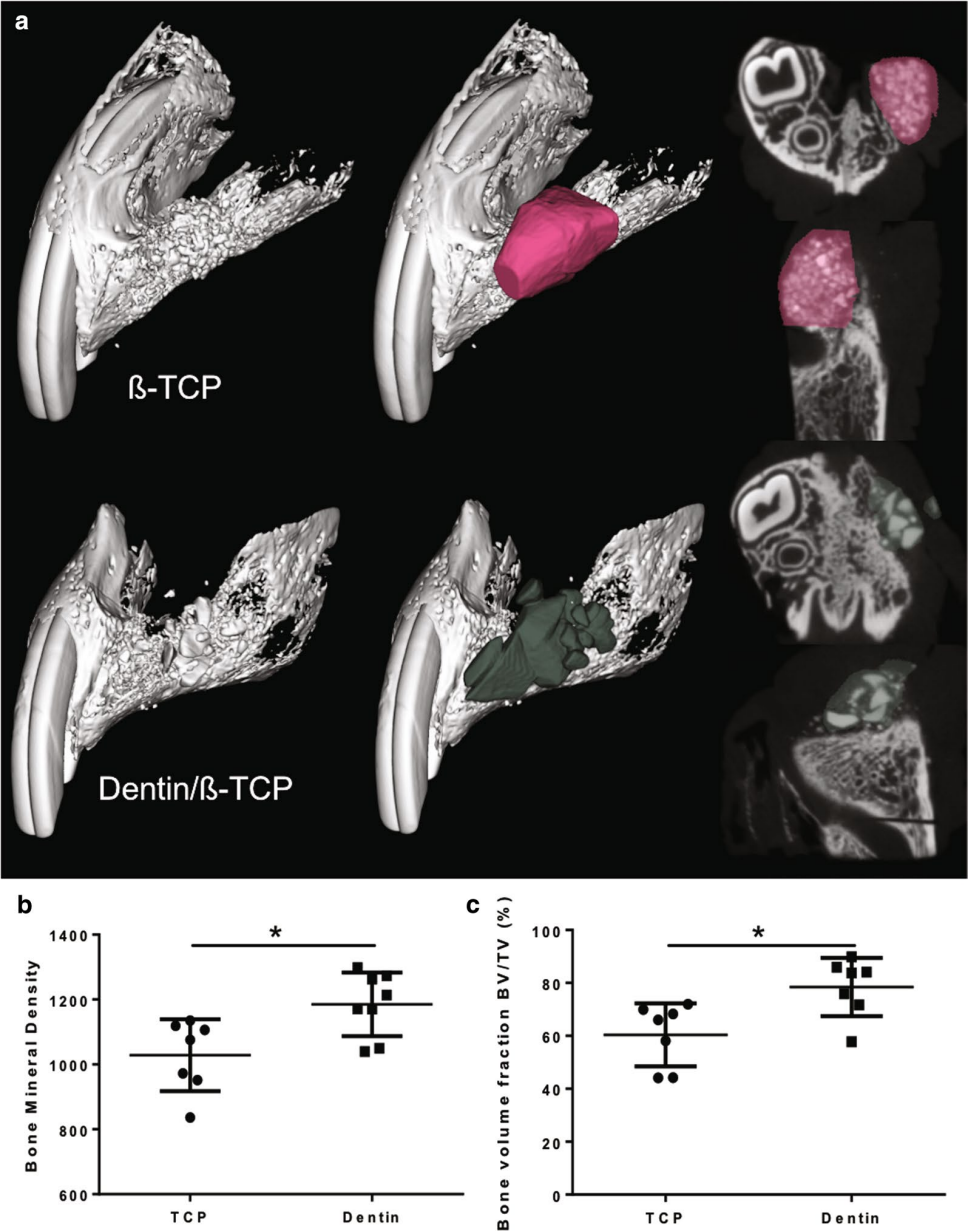
µCT imaging analysis of the repaired alveolar clefts. **a** Healed alveolar cleft 8 weeks after insertion of the grafting intervention with β-TCP and dentin/β-TCP, color outlining the residual volume of the grafted area. **b** Quantitative analysis of bone mineral density (BMD) of defect areas filled with β-TCP or dentin/β-TCP. **c** Bone volume fraction (%) of the bone volume fraction (BV = bone volume/TV = tissue volume) in the defect area of each group. (* indicates p < 0.05)

The results from *µCT imaging* showed that in both defect groups there were bridging of bone and filling of the defects at the 8-week time (Fig. 3). Statistical analysis of the imaging parameters (Table 1) showed that there was significance difference in the Hounsfield unit (HU) between clefts filled with dentin/β-TCP (3455 ± 150.3) versus defects filled with β-TCP (3421 ± 103.2). Furthermore, there was a statistically significant higher amount of percentage bone volume fraction (BV/TV) noted in the composite dentin/β-TCP filled defects by µCT (78.46 ± 4.16) compared to β-TCP group (60.39 ± 4.5), likely representing higher bone formation activity (Fig. 4). The bone mineral density (BMD) comparison correlated also with these radiographic findings with significantly higher values in the composite dentin/β-TCP filled defects (1185 ± 34.68) when compared to the β-TCP filled defects (1028 ± 41.87) (Fig. 4).

**Table 1:** Results of the µCT measurements

	No. of clefts	Defect size (mm^3^)	Hounsfield unit (HU)	% bone volume fraction (BV/TV)	Bone mineral density (BMD)
β-TCP	8	119.84 ± 32.88	3421 ± 103.2	60.39 ± 4.5	1028 ± 41.87
Dentin/β-TCP	8	151.38 ± 29.53	3455 ± 150.3	78.46 ± 4.16	1185 ± 34.68
Mean difference		− 31.54 ± 15.62	33.95 ± 182.3	18.07 ± 6.13	156.8 ± 53.9
p value		0.063		0.0121*	0.0122*
95% CI of MD		− 65.05 to 1.97		4.726 to 31.42	40.4 to 273.30

Mean Hounsfield unit (HU), % bone volume fraction (BV/TV), and bone mineral density (BMD) in defect groups, (means ± standard deviations); CI, confidence interval; MD, mean difference

* Statistically significant

### Histomorphometric analysis

**Fig. 5:**
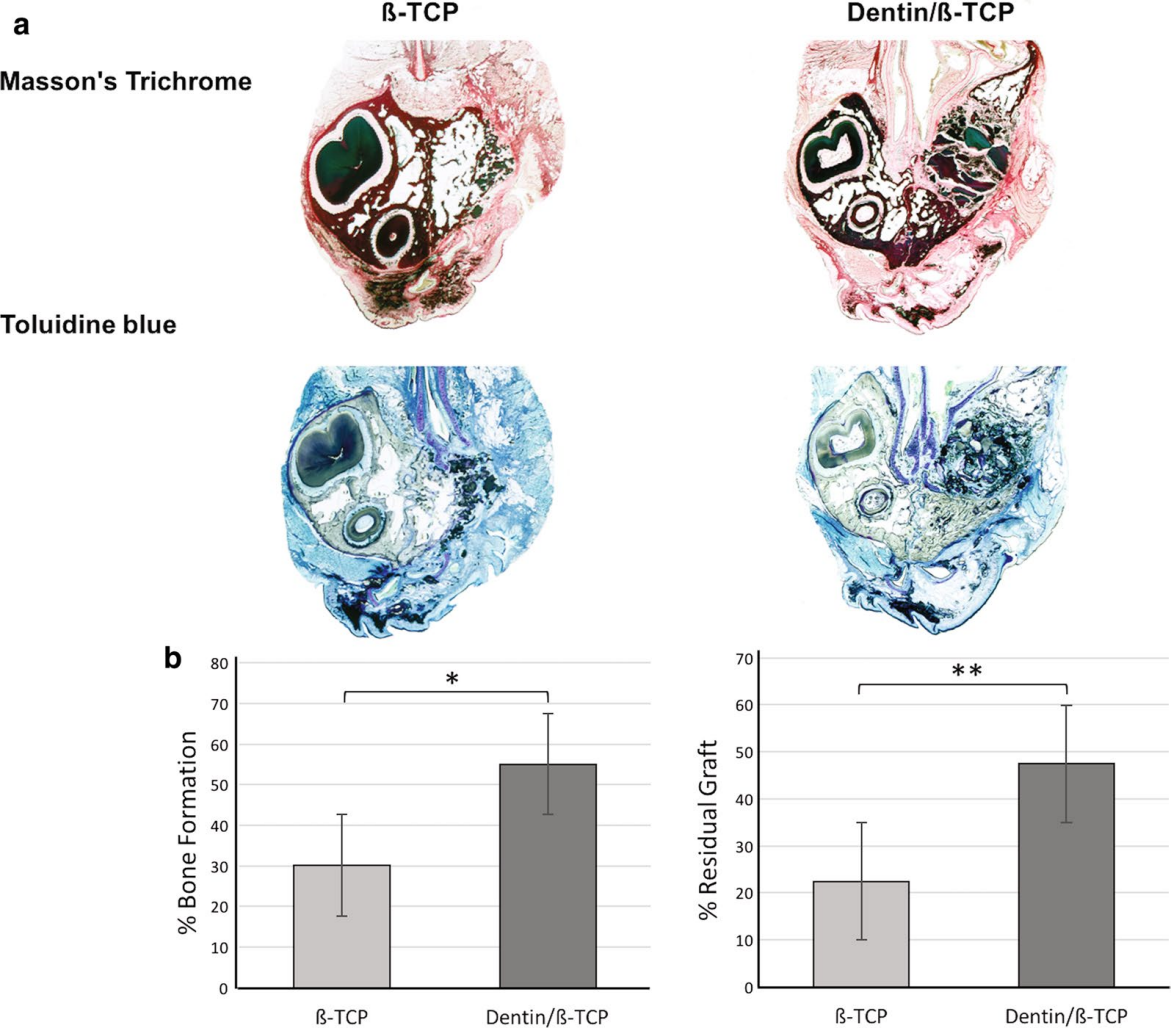
Histological representation of the repaired alveolar clefts. **a** Coronal sections of the repaired alveolar clefts 8 weeks after insertion of the grafting intervention stained with Masson’s trichrome and toluidine blue. **b** Quantitative analysis of percentage bone formation and percentage residual grafts of defect areas filled with β-TCP or dentin/β-TCP. (* indicates p < 0.05, **p < 0.01)

**Fig. 6:**
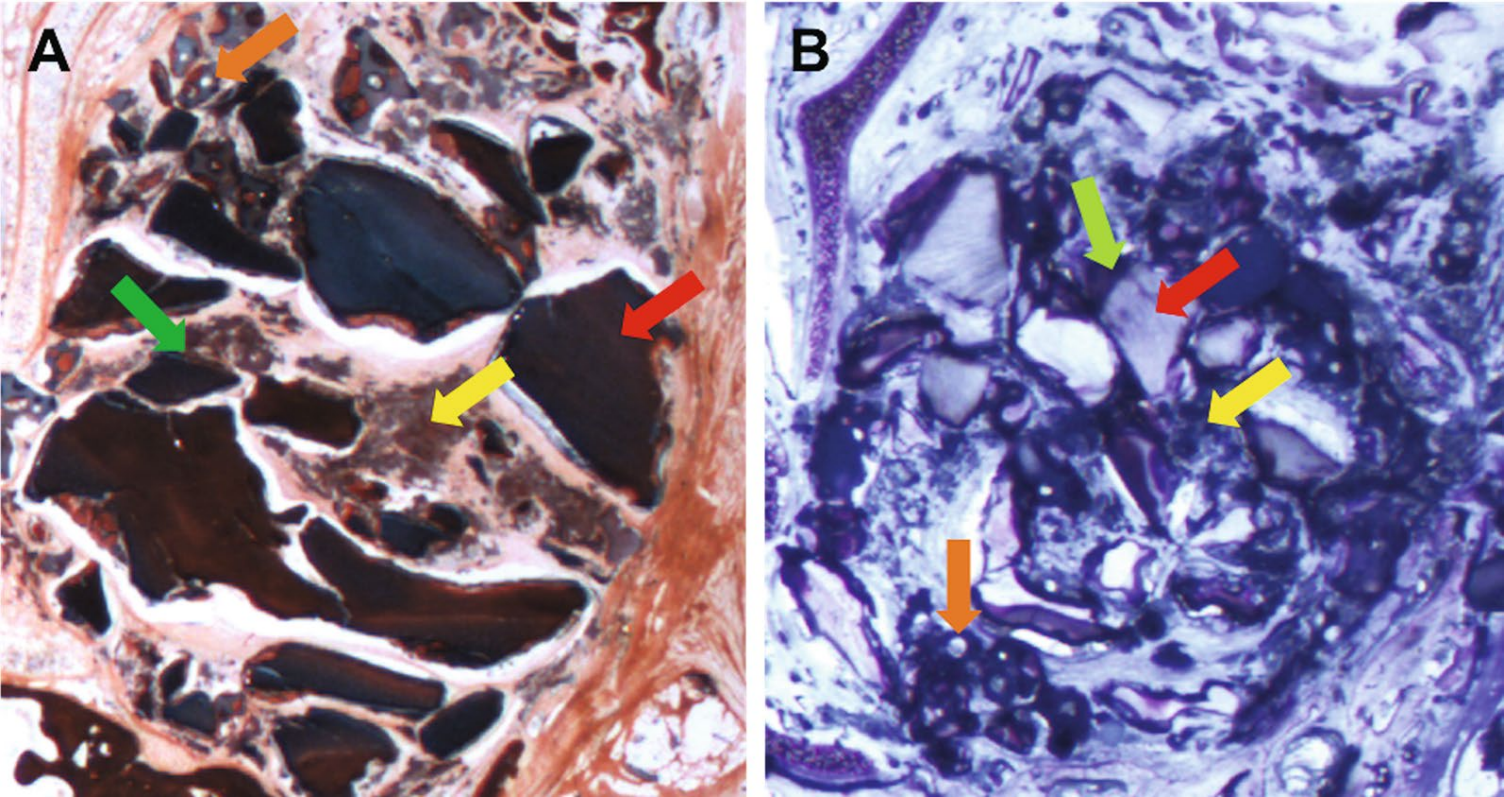
Histological representation of the repaired alveolar clefts. Histological sections of the repaired alveolar clefts 8 weeks after insertion of the grafting intervention stained with Masson’s trichrome (**A**) and toluidine blue (**B**). Red arrow: residual dentin; yellow arrow: newly formed bone; fused dentin with bone; orange arrow: resorption lacunae associated with β-TCP

Histological analysis after 8 weeks showed bone healing pattern which correlated well with the µCT findings. Both β-TCP and dentin/β-TCP filled cavities showed enhanced new bone formation. Histomorphometric analysis showed higher percentage of bone volume fraction (p < 0.05) in dentin/β-TCP group (55.06% ± 7.20) compared to β-TCP group (30.08% ± 9.08) (Table 2, Figs. 5, 6). Percentage residual graft was also significantly higher (p < 0.001) in dentin/β-TCP groups (47.44% ± 6.72) with grossly visible dentin chips compared to the β-TCP group (22.46% ± 2.80), which also showed a lower graft area on gross examination of the slides (Fig. 5). Areas of fused dentin with bone (ankylosis) was noted, and resorption cavities in the dentin were also noted (Fig. 6).

**Table 2:** Results of histomorphometry measurements

	N	% bone formation	% residual graft
β-TCP	8	30.08 ± 9.08	22.46 ± 2.80
Dentin/β-TCP	8	55.06 ± 7.20	47.44 ± 6.72
Mean difference		− 24.98 ± 3.71	− 28.17 ± 2.92
p value		0.0114*	0.0017*
95% CI of MD		− 32.93 to − 17.02	− 37.24 to − 20.73

% bone formation = NBA/TDA × 100 and % residual graft = RGA/TDA in defect groups, (means ± standard deviations)

NBA, new bone area; TDA, total defect area; RGA, residual graft area; CI, confidence interval; MD, mean difference

* Statistically significant

## Discussion

Maxillary alveolar defects are anatomically unique with regard to their overlying soft tissues; the oral mucosa inside the mouth and the nasal mucosa as the nasal floor lining, each with distinct bio-environment. This study demonstrated the feasibility of establishing a reliable skeletal defect in rabbits prior to the later grafting of the defect in a two stage surgery to study alveolar cleft repair healing pattern using various novel biomaterials in three-dimensional form similar in morphology to human patients [50]. Many previous studies in the literature have only filled a created a bone defect in a one stage surgery [45, 47, 53–55], which is not in accordance with closing a cleft clinically where oral and nasal epithelium is lining the defect.

A suitable bone substitute grafting materials for alveolar cleft repair should have osteoinductive and osteoconductive properties, to promote new bone formation, in addition to reliably resorbable scaffold that is subsequently replaced with functionally viable bone that would allow teeth movement into the defect and eruption of teeth [2, 3, 6, 9–12, 56]. The major issue encountered in alveolar cleft repair is the inherent resorption rate associated with autogenous bone grafts, therefore, several studies have focused on attempting to develop new bone substitute materials for bone regeneration that would be less susceptible to bone resorption through several methods including cell therapy with enhanced tissue-engineered scaffolds using mesenchymal stem cells (MSC) or osteoblasts (OB), application of growth factors such as bone morphogenic proteins (BMP), platelet rich plasma (PRP), platelet derived growth factors (PDGF), or various alloplastic biocomposites including calcium phosphate (TCP), hydroxyapatite (HA), or bioglass [4, 8, 11, 12, 56, 57].

Calcium phosphate ceramics are widely utilized in clinical practice as bone substitute materials and contain various forms of alloplastic biomaterials, mostly as calcium sulfate, tricalcium phosphate and the recently introduced biphasic TCP (β-TCP) [58, 59]. β-TCP is available as bone grafting materials in powder granules, blocks, and as injectable paste with nano-micro hydroxyapatite granules to create a non-hardening a moldable mixture, like Maxresorb (Botiss Biomaterials, Berlin, Germany). Calcium phosphate ceramics are often subject to prolong healing time given their lack of osteoinductive properties and their graft volume maintenance depends on the level of resorbability of these alloplastic materials and their replacement rate by bone [59–61]. For modification of the β-TCP resorption properties, a balanced ratio of HA and β-TCP is created to achieve a suitable resorption rate to allow bone formation by replacement, with most studies reporting optimal clinical HA/β-TCP ratio between 65:35 and 55:45 [58, 62–64].

Since HA particles are less susceptible to resorption and acts as structural scaffold for ingrowing osteogenesis and angiogenesis while the dissolution of β-TCP during grafting procedures yields Ca and PO4 ions, which have also been suggested to stimulate bone formation [58, 59, 65]. De Ruiter et al. has compared bone healing with β-TCP and autogenous bone in alveolar cleft repair in a goat model and reported that β-TCP resulted in bone healing similar to iliac crest bone [2]. They reported increased histologic bone formation area in β-TCP group (22.90%, SD 5.62) compared to autogenous iliac crest (20.87%; 5.40); however, no statistical difference was found between the results [2]. A later study by Janssen et al. evaluated microstructured β-TCP granules, embedded in a carboxymethyl cellulose in glycerol (CMCG) putty as alternative grafting material in comparison to autogenous iliac crest bone for alveolar cleft repair in a goat bilateral alveolar cleft model [3]. They reported an improved surgical handling of the β-TCP material during alveolar cleft repair with volumetric µCT and histomorphometric analyses demonstrating integration of both autogenous and β-TCP/CMCG with no significant differences between both groups in percentages of bone formation on the histological sections or reconstructed bone volume with µCT [3].

Previous studies on teeth replantation have demonstrated that delayed replantation of teeth, where the periodontal ligament has become injured and necrotic, e.g. by extensive drying, the dentin will fuse with bone and undergo ankylosis [21, 29–31, 66, 67]. The ankylosed teeth, which made mostly of dentin, undergo gradual resorption process and later go through osseous replacement in a process called replacement resorption where the dentin is replaced by bone [21, 23–25, 29, 30, 66]. This has prompt further investigations into dentin as bone grafting materials. Additional studies have utilized this clinical phenomenon to preserve alveolar ridge height and width through decoronation of tooth or delayed replantation of teeth into the alveolar process to be slowly replaced by newly formed bone, thus maintaining the volume of alveolar process and preventing its resorption [31, 66, 68, 69].

Further experimental studies in rabbits demonstrated the ability of human xenogenic dentin blocks grafts to integrate into native bone and then slowly replaced by new bone with minimal inflammatory reaction [24, 25]. They reported integration of the dentin grafts with bone in 86% of the dentin surface after 3 months and 98% after 6 months. Interesting was their findings of showing continuous resorption of dentin with bony remodelling after 6 months with osseous replacement in the resorption cavities in a gradual process with subsequent replacement of dentin with new bone. Several studies demonstrated the lack of inflammation during healing which can be suggestive of the low level of immunogenicity with dentin [21, 24, 25]. Indicating that this is mere a remodelling resorption process than infective related [29, 66].

Additional studies have shown that human dentin possesses osteoinductive properties, likely explained by its inherent reservoir of bone morphogenic protein (BMP) and its function as a carrier of these growth factors [21, 32, 70, 71]. The osteoinductivity potential of dentin was further investigated in a study by study by Al-Asfour et al. they demonstrated that xenogenic non-demineralized dentin can promote new bone formation when placed in the marrow space of rabbits tibia [21]. In their study, more bone formation was seen on the dentin when the graft was placed close proximity to native bone, suggesting a bigger role of osteoconductive properties of dentin during graft healing in comparison to the osteoinductive properties [21]. Additional work by the same research group demonstrated minimal bone formation when dentin was implanted in non-osteogenic environment suggesting a decrease role osteoinductivity non-demineralized dentin [23].

Mordenfeld et al. investigated the effect of demineralization of xenogenic porcine dentin on bone formation in an osteoconductive environment in a rat model and noted a higher rate of resorption of the dentin grafts with increasing level of graft demineralization [33]. Their results also demonstrated a significant increase in bone formation with increasing the degree of demineralization of the dentin, as long as minimal inflammatory process was present around the dentin graft. This suggest that bone regeneration and healing of the dentin graft was closely related to the inflammatory process during healing phase, and that incorporation of dentin with native bone was seen when inflammation is absent, with subsequent gradual resorption and replacement with bone [21, 24, 25, 33, 61]. Mordenfeld et al. study also demonstrated that grafting dentin resulted in fibrous encapsulation and compromised graft healing when dentin blocks were placed loosely beneath the periosteum of rat skull, likely as a result of the micromovements caused by non-fixation of the dentin graft during bone healing phase. On the other hand, superior graft healing and incorporation into bone was seen when dentin blocks were inserted into a tighter pocket, which restricted the mobility of the graft [33]. These results are consistent with findings from other animal studies which demonstrated that dentin blocks undergo resorption when implanted in a non-osteogenic environment, such as in the muscle or the subcutaneous tissue, due to mobility of the graft leading to fibrous tissue reaction and inability to induce bone formation [21, 23, 34, 72]. For this reason, the stability of the dentin graft, local tissue environment around the graft, and the susceptibility to inflammatory process seems to have an essential role in healing of the dentin grafts and its incorporation into bone [21, 23–25, 31, 33, 34]. Further comparative study of bone regeneration pattern in rabbit’s tibia between autogenous onlay bone blocks and xenogenic demineralized dentin only blocks were conducted by Al-Asfour et al. and demonstrated notable new bone formation through replacement of the integrated dentin at the native bone/dentin graft interphase with similar resorption rate and pattern of the dentin blocks to the autogenous bone over 12 weeks period [22]. By constructing a composite putty β-TCP/HA and dentin blocks mixture, we aimed at improving the handling properties of the dentin blocks during graft insertion into alveolar clefts and restricting dentin grafts mobility, hence achieving better graft stability in the defect to evaluate of the regenerative potential of dentin/β-TCP graft on osteogenesis.

Our results show that alveolar clefts defects filled with composite dentin/β-TCP showed the highest number of bone volume fraction (BV/TV) at (78.46% ± 4.16), and bone mineral density (BMD) (1185 ± 34.68) when compared to the alveolar defects filled with β-TCP alone (Table 1). These differences were statistically different (α < 0.05) suggesting that the composite dentin and β-TCP/HA mixture has successfully healed with the native bone. The higher % bone volume fraction and BMD can be likely attributed to the residual dentin grafts, which show a slower rate of resorption and may possess inherent osteoinductive properties. This correlated well with previous finding reported by Al-Asfour, Andersson, and Mordenfeld groups [21–25, 33]. Our histological findings correlated well with our radiographic findings with composite dentin/β-TCP demonstrating statistically higher (α < 0.05) bone volume fraction (55.06% ± 7.20) compared to β-TCP group (30.08% ± 9.08), suggesting superior overall bone volume formation in the composite group. Of note was the significantly higher percentage residual graft in the in addition to higher percentage of residual grafts in the dentin/β-TCP group (47.44% ± 6.72) compared to the β-TCP group (22.46% ± 2.80) after 8 weeks healing period (Table 2), which is consistent with previous studies reporting slower resorption rate of dentin grafts which leads to better graft volume maintenance [21, 22, 24, 25, 33, 34, 61]. In our study, the β-TCP/HA mixture was non-hardening, and served the purpose of stabilized the dentinal blocks to allow osteogenesis and expression of any osteoinductive properties.

The results of this study demonstrated a reliable alveolar cleft animal model of testing of novel bone substitute materials after successful grafting and bone formation, and that alveolar cleft defects repair with dentin/β-TCP achieved higher graft residual volume and bone volume fraction when evaluated by µCT and histomorphometry.

## Conclusions

Grafting of alveolar cleft defects in rabbits with composite xenogenic dentin with beta-tricalcium phosphate achieved superior bone volume fraction and residual graft volume to clefts repaired with beta-tricalcium phosphate alone when evaluated radiographically and histologically.

## Abbreviations

BMD: bone mineral density
β-TCP: beta-tricalcium phosphate
BV/TV: % bone volume fraction
HA: hydroxyapatite
HU: Hounsfield unit
µCT: micro-CT
NZW: New Zealand White rabbit
SD: standard deviation
